# The contribution of health behaviour to socioeconomic inequalities in alcohol harm: Analysis of the UK biobank, a large cohort study with linked health outcomes

**DOI:** 10.1016/j.ssmph.2023.101443

**Published:** 2023-06-10

**Authors:** Jennifer Boyd, Kate Hayes, Dan Green, Colin Angus, John Holmes

**Affiliations:** a School of Health and Related Research, The University of Sheffield, Sheffield, UK; b MRC/CSO Social and Public Health Sciences Unit, School of Health and Wellbeing, University of Glasgow, Glasgow, UK; c College of Health and Life Sciences, Aston University, B4 7ET, Birmingham, UK

**Keywords:** Alcohol, Inequality, Socioeconomic position, Deprivation, Data linkage, UK Biobank

## Abstract

This is the first study to use the UK Biobank database to: 1) test whether participants of a low socioeconomic position (SEP) are less likely to drink, but more likely to suffer alcohol-related harm, and 2) test the contribution of behavioural factors. The database contains health-related information from 500,000 UK residents that were recruited aged 40–69 between 2006 and 2010. Our analysis focuses on participants resident in England (86% of the total sample). We obtained baseline demographics, survey data regarding alcohol consumption and other behaviours, and linked death and hospital-admission records. The primary outcome was time from study entry to experiencing an alcohol-attributable event (hospital admission or death). The relationship between alcohol-attributable harm and five measures of SEP (area-level deprivation, housing tenure, employment status, household income and qualifications) was investigated using time-to-event analysis. Average weekly alcohol consumption, other drinking behaviours (drinking history and beverage preference), and lifestyle factors (BMI and smoking status) were added incrementally as covariates in nested regression models to investigate whether they could explain the relationship between harm and SEP. 432,722 participants (197,449 men and 235,273 women) were included in the analysis with 3,496,431 person-years of follow-up. Those of a low SEP were most likely to be never/former drinkers or high-risk drinkers. However, alcohol consumption could not explain experiences of alcohol-attributable harm between SEP groups (Hazard Ratio (HR) 1.48; 95% Confidence Interval 1.45–1.51, after adjusting for alcohol consumption). Drinking history, drinking mostly spirits, an unhealthy Body Mass Index and smoking all increased the risk of alcohol-attributable harm. However, these factors only partially explain SEP differences in alcohol harm as the HR for the most deprived vs the least deprived was still 1.28 after adjustment. This suggests that improving wider health behaviour of the most deprived could reduce alcohol-related inequalities. However, a substantial proportion of the variance in alcohol harm remains unexplained.

## Highlights

• Average alcohol consumption alone cannot explain inequalities in alcohol harm.
• Drinking history, beverage type, BMI and smoking partially explain the AHP.
• The HR for the least deprived was 1.26 after adjusting for all health behaviours.
• Explanations (e.g., biological and social determinants) remain untested.

## 1 Introduction

Alcohol consumption contributes significantly to death, disease and disability globally (Griswold et al., 2018; World Health Organization, 2018). However, the burden of alcohol-related harm is not evenly distributed across socioeconomic groups. Rates of both alcohol-related mortality (alcohol as a contributory cause) and alcohol-specific mortality (alcohol as the sole cause) are substantially higher for the most disadvantaged socioeconomic groups compared to the most advantaged (Leyland, Dundas, McLoone, & Boddy, 2007; Mackenbach et al., 2015; Mäkelä & Paljärvi, 2008). This is despite evidence that those in disadvantaged socioeconomic groups consume the same or smaller amounts of alcohol on average when compared to their more advantaged counterparts (Erskine, Maheswaran, Pearson, & Gleeson, 2010; Katikireddi, Whitley, Lewsey, Gray, & Leyland, 2017). This counter-intuitive phenomenon is termed the ‘alcohol harm paradox’ (AHP) and contributes to wider health inequalities (Angus et al., 2020), the reduction of which is a priority within the United Nations' 2030 Agenda for Sustainable Development (United Nations, 2015). Yet the mechanisms which create and sustain the AHP remain unclear.

Cross-sectional research has attempted to characterise differences between socioeconomic groups to provide explanations for the AHP. These studies have tested for differences in drinking patterns, drinking histories, engaging in multiple risk behaviours, and underreporting of alcohol consumption within surveys (Bellis et al., 2016; Lewer, Meier, Beard, Boniface, & Kaner, 2016; Livingston, 2014). The resulting evidence demonstrates that deprivation is related to current and past heavier drinking patterns, and engaging in multiple health risk behaviours such as smoking, eating unhealthily, and having low levels of exercise (Bellis et al., 2016; Lewer et al., 2016). However, these studies do not test the degree to which these health risk behaviours explain differences in alcohol-related harm between people in different socio-economic positions (SEP).

To our knowledge there are three existing studies which employ data linkage methods to investigate the contribution of health risk behaviours to the AHP (Gartner et al., 2019; Katikireddi et al., 2017; Peña et al., 2021). These studies have found that heavier drinking patterns, engaging in multiple unhealthy behaviours (e.g., smoking and poor diet) and beverage type at best only partially attenuate the relationship between socioeconomic position, average alcohol consumption and alcohol-related harm (Gartner et al., 2019; Katikireddi et al., 2017; Peña et al., 2021). In Kattikireddi et al. (2017) the rate of alcohol-attributable mortality and hospital admissions was three times higher for the most disadvantaged compared with the most advantaged and this association remained after adjusting for weekly consumption and heavy drinking patterns, and was only slightly attenuated after further adjusting for smoking and Body Mass Index (BMI) (Katikireddi et al., 2017). However, each of the existing studies has a limitation: small sample size (Gartner et al., 2019), and that they do not investigate the impact of beverage type or drinking history (Katikireddi et al., 2017; Peña et al., 2021). Beverage type is particularly important to consider given that stronger drinks facilitate becoming more drunk quickly and therefore are riskier to consume (Mäkelä, Mustonen, & Österberg, 2007). Additionally, the consumption of stronger alcoholic beverages can be considered riskier as there are some conditions that relate specifically to alcohol intoxication (e.g., injuries and suicide).

Given the limited availability of research investigating the contribution of health risk behaviour to the AHP using linked data, this study aimed to test whether the AHP is observed in a large-scale database linked to health outcomes. More critically this study also aimed to investigate the contribution of other health related factors, specifically drinking history, beverage type, BMI and smoking, to the differences in alcohol-attributable harm between socioeconomic groups. Based on previous literature we hypothesised that these behavioural risk factors would partially contribute to the AHP but would be unable to fully explain the relationship.

## 2 Methods

The UK Biobank is a large-scale database which contains health-related information from 500,000 UK residents who were recruited aged 40–69 between 2006 and 2010. Participation in the Biobank is voluntary and participants were recruited to take part via invitation to attend the assessment centre. Indices of Multiple Deprivation (IMD), an area-based composite measure of deprivation and a crucial measure of SEP in our study, are not constructed to be comparable across nations within the UK. Therefore, we limited our sample to Biobank participants resident in England, approximately 86% of the total (432,729 respondents). IMD was assigned to participants based on participant postcode.

The UK Biobank is a mix of self-reported survey data and linked administrative records. For this study we acquired the baseline self-reported survey data collected at the assessment centre and the subsequent admission and death data for the subsequent years. The dataset offers information on participants current and past drinking habits, as well as broader health-related behaviours. Multiple measures of SEP, both individual-level and small area-level, were recorded. This study can also provide insights into a new context as it is based on an English population sample, while previous papers were focused on Scotland, Wales and Finland (Gartner et al., 2019; Katikireddi et al., 2017; Peña et al., 2021).

### 2.1 Variables

#### 2.1.1 Socioeconomic position

In this study we used multiple SEP measures and repeated our analysis for each measure. SEP is a complex concept that relates to the area a person lives in, their income, education, occupation, housing tenure and employment status, among many other things. Using a number of different measures in our analysis helps capture different dimensions of the effect SEP has on a person's behaviour and health (Beard et al., 2016; Katikireddi et al., 2017). The primary measure of SEP used in this study is the IMD 2010, a composite, small area-level measure of socioeconomic deprivation (GOV UK, 2010). IMD quintiles from 2010 were preferred to more recent iterations as they aligned more closely with the spread of recruitment dates in the sample.

Four individual-level measures of SEP were also constructed. Highest educational achievement was created using the international standard classification of education (ISCED) to categorise participants into five groups: Degree or above; other lower tertiary level qualification (HNC, HND, NVQ, other professional qualification e.g., nursing); A levels/AS levels or equivalent (ISCED level 4); O levels/GCSEs or equivalent e.g., CSEs (ISCED level 3); Other/no school (ISCED levels 0,1 or 2). Household income was categorised into five groups (Less than £18,000; £18,000 to £30,999; £31,000 to £51,999; £52,000 to £100,000; Greater than £100,000). Housing tenure was categorised into 3 groups: homeowners (owned outright, mortgage, shared ownership); renters (rent private landlord, rent from local authority); don't pay (live in accommodation rent free). Employment status was split in two: paid employment (in paid employment or self-employed); and unpaid position (retired, looking after home and/or family, unable to work because of sickness or disability, unemployed, doing unpaid or voluntary work, full or part-time student, or none of the above).

#### 2.1.2 Alcohol consumption

Participants were asked questions regarding whether consumed alcohol at all. Those who did not were asked whether they were former or never drinkers. Those who did were asked about their frequency of consumption; with responses ranging from daily or almost daily to never. Participants with more/less frequent consumption answered further questions regarding their typical weekly/monthly consumption of measures of different types of alcoholic drinks, for example of glasses of wine or pints of beer. We converted these measures into an estimate of their average number of UK units of alcohol (1 unit = 8g of ethanol) consumed weekly with the assumption that monthly consumption was evenly spread across the four weeks of the month. Respondents were then categorised into six groups: never drinkers; ex-drinkers; occasional drinkers (<2 units per week for both men and women); moderate drinkers (<14 units per week for both men and women); increasing risk (14–35 units per week for women, 14–50 units per week for men); and high-risk drinkers (35+ units per week for women, 50+ units per week for men), in line with the current UK Drinking Guidelines (Department of Health, 2016).

#### 2.1.3 Alcohol harm

Hospitalisation or death from an alcohol-related condition as either a primary or secondary diagnosis; was used to capture all deaths and admissions caused by alcohol, but also includes admissions which are not causally linked to alcohol (see Appendix A for the full list of ICD-10 codes).

#### 2.1.4 Other drinking and health behaviours

We investigate the effect of drinking history on future harm by including a variable which captured whether a person had stopped or reduced their alcohol consumption as a result of health concerns. This was assessed using two self-report questions: reasons a participant reduced drinking and reasons a participant stopped drinking. The change in alcohol consumption was considered due to ‘health concerns’ if the participant reported that they reduced or stopped due to illness or ill-health, doctors' advice or health precaution. Preference for type of alcoholic drink was captured by a categorical variable calculated using the data on alcohol consumption described above with the following groups: majority wine (wine constitutes >50% of units consumed); majority beer/cider (beer/cider constitutes >50% of units consumed); majority spirits (spirits constitute >50% of units consumed); mixed consumption (consumption of one type of beverage did not surpass 50% of the units consumed per week). Binge drinking was defined as whether participants typically frequency of consuming six or more units in a single session for both men and women. However, this binge drinking measure had a high percentage of missing values (68.39%); this is because binge drinking questions were only asked to a relatively small subset of the UK Biobank population. Therefore, binge drinking was not included in the main results reported in this paper but can be found in Appendix B.

Current smoking status at the point of recruitment into the study was categorised into four groups: never-smokers, ex-smokers, occasional smokers, current smokers. Respondents’ BMI scores, constructed from height and weight measured during the assessment centre visit, were also categorised: underweight (<18.5), healthy (18.5–25), overweight (25–30), obese (>30).

#### 2.1.5 Other confounders

Three demographic variables (age, sex and ethnicity) were used in the analysis. Age was defined as age at the date of attending an assessment center, truncated to the whole year, and was categorised as follows: <45; 45–49; 50–54; 55–59; 60–64; 65–69; 70+. The variable sex was taken from the participants’ NHS records. When recruited into the study a portion of the participants then updated their sex. The biobank records information on participants self-reported ethnic background, broken down into a number of detailed categories, with small numbers in most categories apart from those from white backgrounds. We therefore collapsed these categories into two: white and non-white.

As the biobank is linked to hospital records, we have information on participants' admission from the years prior to their recruitment to the study. We used this information to define a ‘prior event’ variable, representing whether or not an individual had experienced an alcohol-related event before entering the study, to be included as a confounder in our models. We included the prior event variable as a confounder as we cannot attribute any prior admissions to a level of alcohol consumption as their alcohol consumption prior to their previous admission is not known. Therefore, it is important to include prior event as a confounder to isolate the effect of current alcohol consumption on future alcohol-related admission or death given that previous admission undoubtedly increases the risk of future admission or death and therefore would confound the results.

### 2.2 Data missingness

Missingness was investigated for each variable included in the analysis. Measures of alcohol consumption including consumption level and, in particular, binge drinking had high levels of missing values. Alcohol consumption is a key variable in the analysis. The decision was made not to interpolate missing values, but to use the data available as is. Binge drinking is also thought to be an important confounding variable. We ran the main analysis on the full available sample, not including binge-drinking as a confounder. This analysis is presented in the results section. Additionally, we reran the analysis on the sub-sample of participants for whom information on binge-drinking was available, this time including binge as a confounder; the result of which can be found in Appendix B.

### 2.3 Statistical analysis

The analysis plan for this research project was pre-registered on the OSF: https://mfr.de-1.osf.io/render?url=https://osf.io/rfs8b/?direct%26mode=render%26action=download%26mode=render.

We conducted time-to-event analysis using Cox proportional hazards regression models, with the event being alcohol harm operationalized as the time between recruitment and the participants first alcohol-related hospital admission for or death. The age and BMI variables were categorised to account for non-linearity. Several variables were found to violate the proportionality assumption, and so their effects were allowed to vary stepwise over time. Descriptive analysis shows that low-SEP participants are more likely to have missing values for the binge drinking variable (Table 1). Therefore, the decision was made to run the analysis on the full sample, not including binge as a confounder. The analysis excluding binge drinking as a confounder is presented in the results section. However, as binge drinking is an important confounding variable, and we reran the analysis again on the sub-sample of participants who answered the binge drinking questions, this time including binge as a confounder; the result of which can be found in Appendix B.

**Table 1:** Characteristics of study sample. Split by Index of Multiple Deprivation quintiles.

	Least deprived – 1 n (%)	2 n (%)	3 n (%)	4 n (%)	Most deprived – 5 n (%)	Total n (%)	% missing
**Demographics**							
*Age*							0.0%
<45	11,304 (8.7%)	9145 (9.3%)	8005 (10.2%)	8375 (12.0%)	7549 (13.4%)	44,378 (10.3%)	
45–49	15,551 (12.0%)	11,832 (12.1%)	10,011 (12.7%)	10,157 (14.6%)	8750 (15.5%)	56,301 (13.0%)	
50–54	18,910 (14.6%)	14,239 (14.5%)	11,905 (15.1%)	10,858 (15.6%)	9213 (16.4%)	65,125 (15.1%)	
55–59	23,872 (18.4%)	17,758 (18.1%)	14,291 (18.1%)	12,175 (17.4%)	9591 (17.0%)	77,687 (18.0%)	
60–64	33,667 (26.0%)	25,298 (25.8%)	19,424 (24.6%)	15,658 (22.4%)	11,793 (20.9%)	105,840 (24.5%)	
65–69	25,604 (19.8%)	19,341 (19.7%)	14,825 (18.8%)	12,273 (17.6%)	9180 (16.3%)	81,223 (18.8%)	
70+	665 (0.5%)	535 (0.6%)	414 (0.5%)	311 (0.5%)	243 (0.4%)	2168 (0.5%)	
*Sex*							0.0%
Male	58,560 (45.2%)	44,159 (45.0%)	35,624 (45.2%)	31,991 (45.8%)	27,115 (48.2%)	197,449 (45.6%)	
Female	71,013 (54.8%)	53,989 (55.0%)	43,251 (54.8%)	37,816 (54.2%)	29,204 (51.9%)	235,273 (54.4%)	
*Ethnicity*							0.6%
White	125,739 (97.0%)	94,486 (96.3%)	73,759 (93.5%)	62,895 (90.1%)	48,508 (86.1%)	405,387 (93.7%)	
Non-white	3333 (2.6%)	3210 (3.3%)	4679 (5.9%)	6443 (9.3%)	7179 (12.8%)	24,844 (5.7%)	
**Alcohol consumption**							16.4%
Never	3923 (3.5%)	3414 (4.1%)	3489 (5.3%)	4090 (7.2%)	4430 (10.2%)	19,346 (4.5%)	
Former	3090 (2.8%)	2877 (3.4%)	2724 (4.1%)	3091 (5.4%)	3616 (8.4%)	15,398 (3.6%)	
Occasional	5640 (5.1%)	4986 (6.0%)	4813 (7.3%)	4167 (7.3%)	3181 (7.4%)	22,787 (5.3%)	
Moderate	50,566 (45.5%)	36,132 (43.1%)	27,612 (41.6%)	21,816 (38.2%)	14,371 (33.2%)	150,497 (34.8%)	
Increasing Risk	42,256 (38.0%)	31,401 (37.5%)	23,575 (35.5%)	19,670 (34.4%)	13,823 (32.0%)	130,725 (30.2%)	
High Risk	5722 (5.2%)	4994 (6.0%)	4205 (6.3%)	4330 (7.6%)	3871 (8.9%)	23,122 (5.3%)	
**Drinking behaviour (among current drinkers)**							
Binge drinker	21,385 (17.5%)	14,867 (16.2%)	10,729 (13.7%)	8834 (14.2%)	5023 (10.5%)	60,838 (15.3%)	68.4%
Reduce for health reasons	18,657 (15.3%)	14,912 (16.3%)	12,451 (15.8%)	11,521 (18.5%)	10,030 (21.0%)	67,571 (17.0%)	0.3%
*Beverage Type Preference*							16.4%
No strong preference	6311 (5.2%)	4572 (5.0%)	3722 (4.7%)	3064 (4.9%)	2095 (4.4%)	19,764 (5.0%)	
Majority wine	70,388 (57.5%)	47,959 (52.3%)	34,472 (43.8%)	25,923 (41.6%)	13,815 (28.9%)	192,557 (48.6%)	
Majority beer/cider	22,626 (18.5%)	20,563 (22.4%)	17,829 (22.7%)	16,843 (27.0%)	15,514 (32.5%)	93,375 (23.6%)	
Majority spirits	4859 (4.0%)	4419 (4.8%)	4182 (5.3%)	4153 (6.7%)	3822 (8.0%)	21,435 (5.4%)	
**Drinking behaviour (among non-drinkers)**							
Stopped for health reasons	1557 (22.2%)	1439 (22.9%)	1337 (21.5%)	1487 (20.7%)	1735 (21.6%)	7555 (21.7%)	0.3%
**Lifestyle factors**							
*Smoking status*							0.2%
Never-smoker	76,978 (59.4%)	55,653 (56.7%)	43,038 (54.6%)	35,358 (50.7%)	25,799 (45.8%)	236,826 (54.7%)	
Ex-smoker	27,824 (21.5%)	22,691 (23.1%)	18,862 (23.9%)	17,070 (24.5%)	13,975 (24.8%)	100,422 (23.2%)	
Occasional smoker	19,198 (14.8%)	14,157 (14.4%)	11,084 (14.1%)	9978 (14.3%)	7216 (12.8%)	61,633 (14.2%)	
Current smoker	5419 (4.2%)	5501 (5.6%)	5718 (7.3%)	7171 (10.3%)	8983 (16.0%)	32,792 (7.6%)	
*Body Mass Index (BMI)*							0.6%
Healthy	47,607 (36.7%)	33,136 (33.8%)	24,988 (31.7%)	20,594 (29.5%)	14,215 (25.2%)	140,540 (32.5%)	
Underweight	688 (0.5%)	449 (0.5%)	403 (0.5%)	383 (0.6%)	321 (0.6%)	2244 (0.5%)	
Overweight	55,567 (42.9%)	42,535 (43.3%)	33,565 (42.6%)	28,838 (41.3%)	22,173 (39.4%)	182,678 (42.2%)	
Obese	25,244 (19.5%)	21,540 (22.0%)	19,379 (24.6%)	19,445 (27.9%)	18,939 (33.6%)	104,547 (24.2%)	
**Events**							
Event prior to study entry	19,312 (14.9%)	16,055 (16.4%)	14,368 (18.2%)	13,840 (19.8%)	13,880 (24.7%)	77,455 (17.9%)	0.0%
Event during period of study	34,202 (26.4%)	27,617 (28.1%)	23,327 (29.6%)	21,854 (31.3%)	20,575 (36.5%)	127,575 (29.5%)	0.0%

Note: Numbers do not sum for variables with missing data.

The models were nested as follows: Model A adjusted only for demographic factors age, sex, and ethnicity, as well as having experienced a prior event, Model B adjusted for the variables in Model A in addition to adjusting for alcohol consumption level. Models C adjusted for age, sex, ethnicity, prior event, alcohol consumption and in addition drinking history and beverage type. Finally, Model D adjusted for all of the variables in Models A-C and additionally smoking status and BMI. We tested the extent to which the inclusion of each set of variables in nested models could explain the relationship between SEP and alcohol-related harm.

The analysis was run initially using IMD quintiles as the measure of SEP. We then repeated this process for the four individual level measures of SEP: housing tenure, household income, employment status, and qualifications. In doing this we can explore whether the paradox is evidenced differently across different measures of SEP.

## 3 Results

### 3.1 Sample characteristics and descriptive analysis

The sample included 432,722 people with 3,496,431 person-years of follow-up. 127,575 people experienced an event (26% of women, 33% of men). The sample is predominantly white (93.7%), and includes more women than men (54.4% vs. 45.6%). Table 1 gives a summary of the sample characteristics split by IMD quintile.

Table 1 depicts patterns of alcohol consumption across IMD quintiles in the unadjusted data. More deprived quintiles have higher proportions of both never-/former-drinkers and high-risk drinkers than less deprived quintiles (Never-drinkers/former-drinkers constitute 10.23%/8.35% of quintile 5 vs 3.53%/2.78% of quintile 1, high risk drinkers constitute 8.94% of quintile 5 vs 5.15% of quintile 1). Additionally, the most deprived generally have a greater proportion are smokers, obese and have experienced prior alcohol-related hospitalisation.

### 3.2 Patterns of harm

Results of Cox proportional hazard models are displayed in Table 2. For each model there was an observed stepped gradient in alcohol-related harm across the five IMD quintiles with the most deprived quintile (Q5) experiencing the greatest risk of alcohol-related harm followed by the next most deprived (Q4) and so on, while the least deprived quintile (Q1) had a consistently lower risk of alcohol-related harm.

**Table 2:** Cox proportional hazards model results for each measure of Socioeconomic Position.

	Model A		Model B		Model C		Model D	
	Adjusted for age, sex, ethnicity and prior events		Adjusted for age, sex, ethnicity, prior events and alcohol consumption		Adjusted for age, sex, ethnicity, prior events, alcohol consumption, and other drinking behaviours (stopped drinking for health reasons and beverage type preference)		Adjusted for age, sex, ethnicity, prior events, alcohol consumption, other drinking behaviours and lifestyle factors (smoking status and BMI)	
	HR (95% CI)	p-value	HR (95% CI)	p-value	HR (95%)	p-value	HR (95% CI)	p-value
**Area-based deprivation**								
1 - Least Deprived	1.00	<0.0001	1.00	<0.0001	1.00	<0.0001	1.00	<0.0001
2	1.09 (1.07–1.10)	.	1.09 (1.07–1.10)	.	1.07 (1.05–1.09)	.	1.05 (1.04–1.07)	.
3	1.17 (1.15–1.18)	.	1.15 (1.13–1.18)	.	1.13 (1.11–1.15)	.	1.09 (1.07–1.11)	.
4	1.28 (1.26–1.30)	.	1.25 (1.22–1.27)	.	1.21 (1.19–1.23)	.	1.14 (1.12–1.17)	.
5 - Most deprived	1.54 (1.51–1.57)	.	1.48 (1.45–1.51)	.	1.41 (1.38–1.44)	.	1.28 (1.26–1.31)	.
**Housing tenure**								
Home owners	1.00	<0.0001	1.00	<0.0001	1.00	<0.0001	1.00	<0.0001
Renters	1.59 (1.56–1.62)	.	1.53 (1.50–1.56)	.	1.47 (1.44–1.50)	.	1.35 (1.32–1.38)	.
Live in accommodation rent free	1.11 (1.04–1.19)	0.002	1.11 (1.03–1.19)	0.005	1.11 (1.03–1.20)	0.004	1.07 (0.99–1.15)	0.079
**Employment status**								
Paid employment	1.00	<0.0001	1.00	<0.0001	1.00	<0.0001	1.00	<0.0001
Unpaid position	1.27 (1.26–1.29)	.	1.23 (1.21–1.25)	.	1.21 (1.19–1.23)	.	1.20 (1.18–1.21)	.
**Household income**								
Greater than 100,000	1.00	<0.0001	1.00	<0.0001	1.00	<0.0001	1.00	<0.0001
52,000 to 100,000	1.18 (1.13–1.22)	.	1.18 (1.14–1.23)	.	1.17 (1.12–1.21)	.	1.14 (1.08–1.17)	.
31,000 to 51,999	1.35 (1.30–1.40)	.	1.34 (1.29–1.39)	.	1.31 (1.26–1.36)	.	1.26 (1.21–1.30)	.
18,000 to 30,999	1.53 (1.47–1.58)	.	1.50 (1.44–1.56)	.	1.44 (1.39–1.50)	.	1.37 (1.32–1.42)	.
Less than 18,000	1.86 (1.79–1.93)	.	1.79 (1.72–1.86)	.	1.69 (1.63–1.76)	.	1.55 (1.50–1.62)	.
**Qualifications**								
Degree or above	1.00	<0.0001	1.00	<0.0001	1.00	<0.0001	1.00	<0.0001
HNC, HND, NVQ	1.28 (1.25–1.30)	.	1.25 (1.23–1.28)	.	1.22 (1.19–1.24)	.	1.14 (1.12–1.17)	.
A levels/AS levels or equivalent	1.11 (1.09–1.13)	.	1.10 (1.08–1.13)	.	1.09 (1.07–1.12)	.	1.06 (1.03–1.08)	.
O levels/GCSEs or equivalent	1.22 (1.20–1.24)	.	1.20 (1.18–1.22)	.	1.17 (1.15–1.19)	.	1.11 (1.09–1.13)	.
No School	1.50 (1.48–1.53)	.	1.46 (1.43–1.48)	.	1.38 (1.36–1.41)	.	1.27 (1.25–1.30)	.

Note: HR = Hazard Ration; CI = confidence interval.

Model A estimates alcohol-related harm with controls for age, sex, and ethnicity. We find that the hazard of experiencing alcohol-related harm as measured by alcohol-attributable death or admission increases monotonically as one moves from the least to most deprived quintile (Q5 vs Q1 Hazard Ratio (HR) is 1.55; 95% Confidence Interval (CI) 1.51–1.57).

**Fig. 1:**
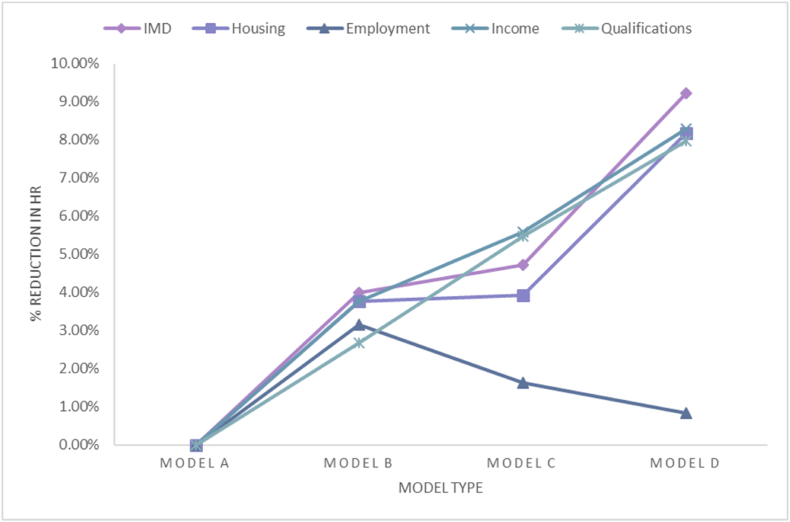
Percentage reduction in Hazard Ratio for each measure of SEP across all four models. *Note: percentages were calculated incrementally so the graph shows the % reduction for Model B compared to Model A, for Model C compared to Model B and for Model D compared to Model C.

Model B estimates alcohol-related harm when additionally controlling for average weekly alcohol consumption. This explains only 4% of this elevated risk of by alcohol-attributable death or admission (HR 1.48; 95% CI 1.45–1.51); see Fig. 1.

Models C and D test explanations for the alcohol harm paradox by controlling for a variety of drinking behaviours and lifestyle factors in addition to demographics and average weekly alcohol consumption. Model C estimates alcohol-related harm controlling for drinking history, beverage type and binge drinking. Drinking history, specifically whether a person has stopped or reduced their alcohol consumption as a result of health concerns significantly increased risk of alcohol-related harm. Drinking mostly wine decreases risk, and drinking mostly spirits is associated with increased risk of alcohol-related harm. Surprisingly, binge drinking is associated with a lower risk of harm after controlling for weekly consumption. Adjusting for these drinking behaviours further reduced the risk associated with being in the fifth IMD quintile as opposed to the first by 4.7% compared to Model B (see Fig. 1).

In the final model, the expected hazard for those in quintile 5 relative to quintile 1 remains 1.28 times higher (95% CI 1.26–1.31). People who are underweight, overweight, or obese have elevated hazards compared to those in the ‘healthy’ BMI range. Being an ex-, occasional, or current smoker increases risks in comparison to being a never-smoker. Adjusting for these additional behavioural risk factors further reduced the risk associated with being in the fifth IMD quintile as opposed to the first by 9.2% compared to Model C.

Magnitudes of effect are broadly similar across all SEP measures. The widest gap is between households earning less than £18,000 a year compared to those earning more than £100,000. Even in the fully adjusted model, hazards are more than 50% higher in low income households (95% CI 1.50–1.62). Therefore, based on this study behavioural factors cannot explain the relationship between SEP and harm for any of the included SEP measures.

### 3.3 Sensitivity analyses

Appendix B provides results for the Cox PH models using the sub-sample with non-missing values for binge drinking and including binge drinking as a confounder. Results follow a broadly similar pattern as in the main analysis, with the inclusion of consumption level, drinking behaviours and lifestyle factors attenuating but not fully explaining the relationship between SEP and harm.

## 4 Discussion

The relationship between SEP, alcohol consumption and alcohol-related harm is complicated in the UK Biobank dataset. Lower SEP groups are similarly likely to exceed the guidelines as those of a high SEP, but those who do so are more likely to drink at higher rather than increasing risk levels. Cox proportional hazard models supported the existence of the AHP, as controlling for alcohol consumption only explained 2.7–4.0% of the increased risk experienced by the lowest SEP across all measures. Therefore, the relationship between SEP and alcohol-related harm appears largely unaffected by differences in average weekly consumption. We also aimed to investigate the extent to which health-related risk behaviours contribute to the excess risk experienced by those of a lower SEP. The results supported our hypothesis that health related behaviours only partially explain the AHP, as adjusting for alcohol consumption, drinking history, beverage type, BMI, and smoker status at most reduces the risk for the most deprived quintile by 5.6–18.0% across all included measures of SEP. While some of the discrepancy in harm across SEP has been explained but the majority persists.

Our findings are consistent with previous record linkage studies (Gartner et al., 2019; Katikireddi et al., 2017; Peña et al., 2021) which also found that the most deprived group consistently face an increased risk of alcohol-attributable outcomes which remains after considering alcohol consumption, BMI and smoking. In terms of beverage type our results closely align with those found by Gartner and colleagues (Gartner et al., 2019). Although beverage type could not explain inequalities in alcohol-related harm, we similarly observed differences in risk of harm by type of beverage. Drinking mostly spirits had the highest increase in risk of alcohol-attributable harm, while drinking mostly wine decreased risk. There is evidence to suggest that the consumption of spirits is associated with binge drinking and needing “*to get really drun*k” (Mäkelä et al., 2007). The difference in risk associated with beverage type could equally relate other characteristics of the type of people drinking those beverages. For example, high socioeconomic groups are purported to drink alcohol in small amounts, frequently and with meals (Degerud et al., 2018). In contrast lower socioeconomic groups drink more heavily per occasion and consume more spirits where larger volumes of alcohol can be consumed quickly (Marmot, 2001). Further research is required to unpick the direct and indirect effects of beverage type on the AHP.

Our analysis also provides a novel insight into potential causes of the paradox by examining drinking history. We found that people who had stopped drinking or had stopped due to a health condition were at greater risk of alcohol-related harm. This supports the explanation that while the most deprived may have reduced their consumption at time of measurement, they still have an increased susceptibility to harm due to their previous alcohol consumption (Boyd, Sexton, et al., 2021).

Critically, this study supports previous research to suggest that behavioural factors play a role in the AHP but cannot fully explain the relationship between SEP and alcohol-related harm (Boyd, Sexton, et al., 2021; Katikireddi et al., 2017; Probst, Kilian, Sanchez, Lange, & Rehm, 2020). There are many other potential explanations for socioeconomic inequalities in alcohol harm including individual level factors (e.g., biological and psychological factors), those associated with the lived experience of disadvantage (e.g., having fewer resources, poorer quality housing and worse access to healthcare), contextual explanations (e.g., living and drinking in more dangerous environments) and impacts from upstream (e.g., alcohol policy and socio-political structures) (Boyd, Sexton, et al., 2021) which could not be tested in this study. Subsequent work has suggested that in order to gain insight into the causal mechanism underlying the AHP we need to utilise existing theories of health inequality (Boyd, Bambra, Purshouse, & Holmes, 2021) which will allow us to design studies with the ability to begin to test some of these neglected explanations for the AHP.

### 4.1 Strengths & limitations

This is the first study to our knowledge that has tested whether behavioural explanations, specifically alcohol consumption, drinking history, beverage type, binge drinking, smoking and BMI, can explain the existence of the AHP observed in England using linked cohort data. We analysed a large dataset with good statistical power and therefore the ability to provide accurate results regarding the observed population. We also used a breadth of SEP measures including area-level deprivation (IMD), housing, employment, income and qualifications, and results were broadly consistent across these measures (with the exception of employment).

There are some limitations to this study that should be noted. Firstly, the income measure that we used did not account for household size and therefore may not accurately reflect the level of wealth of participants given that larger households receiving the same household income as small household will ultimately have less disposable income. However, household income was just one of the five measures of SEP investigated in this study. Secondly, participation is voluntary, and the sample is therefore not necessarily representative. In particular participants in the UK Biobank are more likely to be older (40+ only), female and live in less deprived areas than non-responders and there is evidence to suggest a “healthy volunteer” selection bias (Fry et al., 2017). The underrepresentation of those from more deprived groups and the overrepresentation of older participants is particularly problematic. We also chose to focus on the English sub-population in this study and therefore the results are not representative of the UK. However, the lower proportion of those in the most deprived quintile is not a grave concern given the large sample size and in absolute terms the dataset includes 56,000 participants from the most deprived IMD quintile. The presence of an older population does however limit the events observed to a later alcohol harm trajectory - the primary outcome of alcohol attributable events occurs at age 40 and above (Office for National Statistics, 2021). We have attempted to overcome this limitation by including drinking history, specifically questions on reducing alcohol consumption and stopping drinking for health reasons and including prior alcohol-related events in our analysis. However, it is also important to note that those drinking most heavily may not be included in the study sample as they may be institutionalized or homeless.

Another limitation of this study is the cross-sectional nature of the behavioural data. Measurement of alcohol consumption at baseline is effectively a proxy for lifetime consumption which is common to most epidemiological studies on alcohol-related health risks (Britton, Ben-Shlomo, Benzeval, Kuh, & Bell, 2015). The absence of longitudinal data on drinking or other health risk behaviours could underestimate the true impact of risk behaviours on alcohol-related harm. This is particularly relevant as evidence emphasises the role of cumulative lifetime consumption on the development of liver cirrhosis, a key alcohol attributable harm (Lelbach, 1975; Rehm et al., 2021). Additionally, one study which did explore the cumulative role of health behaviours (smoking, alcohol consumption, diet and physical activity) over the life course found that adjusting for these behaviours attenuated the risk of mortality for those in manual occupations by 38–77% (Whitley, Batty, Hunt, Popham, & Benzeval, 2014). However, longitudinal, and to an extent cross-sectional, datasets which contain behavioural and socioeconomic position measures, and crucially are linked to morbidity and mortality outcomes are scarce. Therefore, it is valuable to utilise datasets which do contain linked harm outcomes particularly as there is some evidence to suggest that recent alcohol consumption rather than in early life has a greater association with the development of alcohol cirrhosis (Askgaard, Grønbæk, Kjær, Tjønneland, & Tolstrup, 2015).

Finally, there were issues with missing data in the dataset which may be cause for concern. Particularly 16.37% of the alcohol consumption data was missing from the dataset. We also had to exclude variables on drug use and physical activity from the analysis due to high missingness (>50%). Therefore, we were unable to test the contribution of drug use and physical activity to the relationship between SEP and alcohol-related harm.

### 4.2 Future directions

The UK Biobank is a unique and rich data resource which has the potential to test many of the proposed explanations for the AHP. Establishing the existence of the AHP in this dataset paves the way for future research attempting to understand its causes to utilise this resource. Specifically, unique to the dataset is the availability of detailed biological data. It has been proposed that the AHP exists due to genetic differences between SEP groups or that engaging in multiple risk behaviours leads to biological alterations which increase susceptibility to harm (Boyd et al., 2021). These explanations remain untested and the UK Biobank offers the opportunity to investigate biological mechanisms underlying the AHP.

It is also clear from this study, and other record linkage studies investigating the AHP, that health related risk behaviours only partially contribute to the excess harm experienced by the most deprived (Gartner et al., 2019; Katikireddi et al., 2017; Peña et al., 2021). This further enforces the idea that future research needs to look at alternative explanations for the AHP. A recent systematic review identified that much of the empirical evidence focused on health risk behaviours, however there were several hypothetical explanations that remain untested (Boyd, Sexton, et al., 2021). In addition to biological mechanisms we still do not understand how stress, unequal availability of resources, the psychosocial experience of deprivation and the accumulation of these experiences overtime, among many other explanations, play a role in the AHP. Therefore, future research should consider a move from behavioural explanations alone to other broader determinants associated with socioeconomic inequality.

## 5 Conclusions

The AHP does exist in the UK Biobank dataset and health-related behaviours are only shown to play a partial role in the excess harm experienced by lower socioeconomic groups. The dataset used in this study is a rich data resource which could be utilised to explore other potential explanations for the AHP given the availability of detailed biological data. Alternatively, future research may choose to explore broader determinants (e.g., unequal availability of resources) associated with socioeconomic deprivation which may directly or indirectly explain the increased harm experienced by the most deprived.

## Ethical statement

Ethical approval was not required as analysis was conducted on an existing data source the UK Biobank.

## Author statement of contribution

**Jennifer Boyd:** Conceptualisation; formal analysis; investigation; methodology; supervision; visualization; writing - original draft; writing - review and editing. **Kate Hayes:** Data curation; formal analysis; investigation; visualization; writing – original draft. **Colin Angus:** Methodology; supervision; writing – review & editing. **Dan Green:** Methodology; supervision; writing – review & editing. **John Holmes:** Conceptualisation; methodology; supervision; writing – review & editing.

## Declaration of competing interest

None.

## Figures and Tables

**Table:** 

Health Outcome	ICD-10 Codes
Alcoholic Liver Disease	K700, K701, K702, K703, K704, K709
Alcoholic Cardiomyopathy, Alcoholic Gastritis, Chronic Pancreatitis, Pseudo Cushings Syndrome Alcohol Induced	I426 K292 K852 K860 E244
Mental and Behavioural Disorder due to use of alcohol	F101 F102 F103 F104 F105 F106 F107 F108 F109
Degeneration, Alcoholic Polyneuropathy, Alcoholic Myopathy, Maternal care for damage to foetus from alcohol	G312 G621 G721 O354
Oropharyngeal cancer	C00 C01 C02 C03 C04 C05 C06 C09 C10 C12 C13 C14
Esophageal, colorectal, liver and bile ducts, pancreatic, larangeal, breast cancer	C15 C18 C19 C20 C22 C25 C32 C50
Hypertensive diseases	I10 I11 I12 I13 I14
Ischaemic heart disease	I20 I21 I22 I23 I24 I25
Cardiac arythmias	I47 I48 I49
Haemorrhagic stroke, ischaemic stroke	I60 I61 I62 I63 I64 I65 I66 I67
Cirrhosis of the liver	K705 K706 K707 K708 K73 K74
Acute pancreatitis	K850 K851 K854 K855 K856 K857 K858 K859
Chronic pancreatitis	K861 K862 K863 K864 K865 K866 K867 K868 K869
Diabetes type 2, epilepsy and status epilepticus	E11 G40 G41
Tuberculosis	A15 A16 A17 A18 A19
Lower respiratory tract infection	J10 J11 J12 J13 J14 J15 J16 J17 J18 J09
Excessive blood alcohol level, toxic effect of alcohol	R780 T510 T511 T518 T519
Alcohol poisoning, evidence of alcohol involvement, acute intoxication	X45 X65 Y15 Y90 F100
Accidental poisoning by exposure to noxious substances	X40 X41 X42 X43 X44 X46 X47 X48 X49 Y10 Y11 Y12 Y13 Y14 Y16 Y17 Y18 Y19 T36 T37 T38 T39 T40 T41 T42 T43 T44 T45 T46 T47 T48 T49 T50 T52 T53 T54 T55 T56 T57 T58 T59 T60 T61 T62 T63 T64 T65
Transport injuries	V01 V02 V03 V04 V05 V06 V07 V08 V09 V10 V11 V12 V13 V14 V15 V16 V17 V18 V19 V20 V21 V22 V23 V24 V25 V26 V27 V28 V29 V30 V31 V32 V33 V34 V35 V36 V37 V38 V39 V40 V41 V42 V43 V44 V45 V46 V47 V48 V49 V50 V51 V52 V53 V54 V55 V56 V57 V58 V59 V60 V61 V62 V63 V64 V65 V66 V67 V68 V69 V70 V71 V72 V73 V74 V75 V76 V77 V78 V79 V80 V81 V82 V83 V84 V85 V86 V87 V88 V89 V90 V91 V92 V93 V94 V95 V96 V97 V98 Y850
Fall injuries	W00 W01 W02 W03 W04 W05 W06 W07 W08 W09 W10 W11 W12 W13 W14 W15 W16 W17 W18 W19
Exposure to mechanical forces	W20 W21 W22 W23 W24 W25 W26 W27 W28 W29 W30 W31 W32 W33 W34 W35 W36 W37 W38 W39 W40 W41 W42 W43 W44 W45 W46 W47 W48 W49 W50 W51 W52
Drowning	W65 W66 W67 W68 W69 W70 W71 W72 W73 W74 Y21
Fire injuries	X00 X01 X02 X03 X04 X05 X06 X07 X08 X09 Y26
Other unintentional injuries	W75 W76 W77 W78 W79 W80 W81 W82 W83 W84 W85 W86 W87 W88 W89 W90 W91 W92 W93 W94 W95 W96 W97 W98 W99 X10 X11 X12 X13 X14 X15 X16 X17 X18 X19 X20 X21 X22 X23 X24 X25 X26 X27 X28 X29 X30 X31 X32 X33 Y20 Y22 Y23 Y24 Y25 Y27 Y28 Y29 Y31 Y32 Y33 Y34
Intentional self-harm	X60 X61 X62 X63 X64 X66 X67 X68 X69 X70 X71 X72 X73 X74 X75 X76 X77 X78 X79 X80 X81 X82 X83 X84 Y870
Assault and other intentional injuries	X85 X86 X87 X88 X89 X90 X91 X92 X93 X94 X95 X96 X97 X98 X99 Y00 Y01 Y02 Y03 Y04 Y05 Y06 Y07 Y08 Y09 Y871 Y35

**Note this list was taken from existing work on the Sheffield Alcohol Policy Model:https://figshare.shef.ac.uk/articles/journal_contribution/Alcohol-attributable_diseases_and_dose-response_curves_for_the_Sheffield_Alcohol_Policy_Model_version_4_0/6819689/2.

## Data Availability

The authors do not have permission to share data.

## Appendix B Supplementary data

Multimedia component 1 Multimedia component 1

Multimedia component 2 **Appendix C:** Characteristics of study sample for each measure of SEP (excel document). Multimedia component 2

Multimedia component 3 **Appendix D:** Full model results for area-level deprivation measure. Multimedia component 3
