# Supplementary material for: The contribution of health behaviour to socioeconomic inequalities in alcohol harm: Analysis of the UK biobank, a large cohort study with linked health outcomes
Source: SSM Popul Health. 2023 Jun 10;23:101443. doi: 10.1016/j.ssmph.2023.101443 (PMC10275713; doi:10.1016/j.ssmph.2023.101443)
Supplement: Multimedia component 1 [file mmc1.docx]

**Appendix B:** Results of the Cox PH models using the sub-sample with non-missing values for binge and including binge as a confounder.

|  | **Model A** | | **Model B** | | **Model C** | | **Model D** | |
| --- | --- | --- | --- | --- | --- | --- | --- | --- |
|  | Adjusted for age, sex, ethnicity and prior events | | Adjusted for age, sex, ethnicity, prior events and alcohol consumption | | Adjusted for age, sex, ethnicity, prior events, alcohol consumption, and other drinking behaviours (stopped drinking for health reasons and beverage type preference) | | Adjusted for age, sex, ethnicity, prior events, alcohol consumption, other drinking behaviours and lifestyle factors (smoking status and BMI) | |
|  | **HR (95% CI)** | **p-value** | **HR (95% CI)** | **p-value** | **HR (95%)** | **p-value** | **HR (95% CI)** | **p-value** |
| **Area-based deprivation** | | | | | | | | |
| 1 - Least Deprived | 1.00 | <0.0001 | 1.00 | <0.0001 | 1.00 | <0.0001 | 1.00 | <0.0001 |
| 2 | 1.06 (1.03-1.09) | .. | 1.07 (1.03-1.10) | .. | 1.06 (1.03-1.09) | 0.0004 | 1.04 (1.01-1.08) | 0.01 |
| 3 | 1.11 (1.07-1.15) | .. | 1.11 (1.07-1.15) | .. | 1.09 (1.06-1.13) | <0.0001 | 1.06 (1.02-1.10) | 0.001 |
| 4 | 1.17 (1.13-1.21) | .. | 1.15 (1.10-1.19) | .. | 1.13 (1.13-1.18) | .. | 1.08 (1.04-1.12) | 0.0002 |
| 5 - Most deprived | 1.36 (1.31-1.42) | .. | 1.31 (1.25-1.38) | .. | 1.28 (1.22-1.34) | .. | 1.18 (1.12-1.24) | <0.0001 |
| **Housing tenure** |  |  |  |  |  |  |  |  |
| Home owners | 1.00 | <0.0001 | 1.00 | <0.0001 | 1.00 | <0.0001 | 1.00 | <0.0001 |
| Renters | 1.43 (1.36-1.50) | .. | 1.34 (1.27-1.41) | .. | 1.31 (1.24-1.38) | .. | 1.18 (1.14-1.21) | .. |
| Live in accommodation rent free | 0.97 (0.84-1.13) | 0.713 | 0.98 (0.84-1.15) | 0.838 | 0.98 (0.84-1.16) | 0.85 | 1.03 (0.99-1.07) | 0.131 |
| **Employment status** | | | | | | | | |
| Paid employment | 1.00 | <0.0001 | 1.00 | <0.0001 | 1.00 | <0.0001 | 1.00 | <0.0001 |
| Unpaid position | 1.13 (1.10-1.16) | .. | 1.11 (1.08-1.14) | .. | 1.10 (1.07-1.13) | .. | 1.10 (1.07-1.13) | .. |
| **Household income** |  |  |  |  |  |  |  |  |
| Greater than 100,000 | 1.00 | <0.0001 | 1.00 | <0.0001 | 1.00 | <0.0001 | 1.00 | <0.0001 |
| 52,000 to 100,000 | 1.15 (1.08-1.22) | .. | 1.15 (1.08-1.22) | .. | 1.13 (1.07-1.20) | .. | 1.11 (1.04-1.18) | .. |
| 31,000 to 51,999 | 1.24 (1.17-1.31) | .. | 1.23 (1.16-1.30) | .. | 1.20 (1.14-1.28) | .. | 1.16 (1.10-1.23) | .. |
| 18,000 to 30,999 | 1.35 (1.27-1.43) | .. | 1.32 (1.24-1.40) | .. | 1.28 (1.20-1.35) | .. | 1.22 (1.15-1.30) | .. |
| Less than 18,000 | 1.54 (1.46-1.64) | .. | 1.48 (1.39-1.58) | .. | 1.42 (1.33-1.51) | .. | 1.32 (1.24-1.41) | .. |
| **Qualifications** |  |  |  |  |  |  |  |  |
| Degree or above | 1.00 | <0.0001 | 1.00 | <0.0001 | 1.00 | <0.0001 | 1.00 | <0.0001 |
| HNC, HND, NVQ | 1.20 (1.16-1.25) | .. | 1.20 (1.15-1.24) | .. | 1.17 (1.13-1.22) | .. | 1.10 (1.06-1.15) | .. |
| A levels / AS levels or equivalent | 1.08 (1.04-1.12) | .. | 1.06 (1.02-1.10) | 0.003 | 1.06 (1.01-1.10) | 0.007 | 1.02 (0.98-1.06) | 0.26 |
| O levels / GCSEs or equivalent | 1.18 (1.15-1.21) | .. | 1.15 (1.11-1.19) | <0.0001 | 1.13 (1.10-1.17) | <0.0001 | 1.07 (1.03-1.10) | <0.0001 |
| No School | 1.34 (1.29-1.39) | .. | 1.32 (1.26-1.38) | .. | 1.27 (1.22-1.33) | .. | 1.17 (1.12-1.23) | .. |
| ***Note: HR = Hazard Ration; CI = confidence interval*** | | | | | | | | |
